# *In Situ* Quantification of Experimental Ice Accretion on Tree Crowns Using Terrestrial Laser Scanning

**DOI:** 10.1371/journal.pone.0064865

**Published:** 2013-05-31

**Authors:** Charles A. Nock, David Greene, Sylvain Delagrange, Matt Follett, Richard Fournier, Christian Messier

**Affiliations:** 1 Center for forest research, Department des Sciences Biologique, Université du Québec à Montréal, Montréal, Québec, Canada; 2 Department of Geography, Planning and Environment, Concordia University, Montréal, Québec, Canada; 3 Institut des Sciences de la Forêt Tempérée, Département des Sciences Naturel, Université du Québec en Outaouais, Ripon, Québec, Canada; 4 Centre d’Applications et de Recherches en Télédétection, Département de Géomatique appliquée, Université de Sherbrooke, Sherbrooke, Québec, Canada; The Ohio State University, United States of America

## Abstract

In the eastern hardwood forests of North America ice storms are an important disturbance event. Ice storms strongly influence community dynamics as well as urban infrastructure via catastrophic branch failure; further, the severity and frequency of ice storms are likely to increase with climate change. However, despite a long-standing interest into the effects of freezing rain on forests, the process of ice accretion and thus ice loading on branches remains poorly understood. This is because a number of challenges have prevented *in situ* measurements of ice on branches, including: 1) accessing and measuring branches in tall canopies, 2) limitations to travel during and immediately after events, and 3) the unpredictability of ice storms. Here, utilizing a novel combination of outdoor experimental icing, manual measurements and terrestrial laser scanning (TLS), we perform the first *in situ* measurements of ice accretion on branches at differing heights in a tree crown and with increasing duration of exposure. We found that TLS can reproduce both branch and iced branch diameters with high fidelity, but some TLS instruments do not detect ice. Contrary to the expectations of ice accretion models, radial accretion varied sharply within tree crowns. Initially, radial ice accretion was similar throughout the crown, but after 6.5 hours of irrigation (second scanning) radial ice accretion was much greater on upper branches than on lower (∼factor of 3). The slope of the change in radial ice accretion along branches increased with duration of exposure and was significantly greater at the second scanning compared to the first. We conclude that outdoor icing experiments coupled with the use of TLS provide a robust basis for evaluation of models of ice accretion and breakage in tree crowns, facilitating estimation of the limiting breaking stress of branches by accurate measurements of ice loads.

## Introduction

Ice storms are one of the most frequent and severe natural disturbances in the deciduous forests of northeastern North America and thus play a central role in forest dynamics. Developing when warm moist air passes over a colder ground-level air mass [1], ice accumulates as rain strikes branches that are at or below freezing. Understanding the biomechanical response of trees to ice loading is important not only because ice storms significantly affect forest dynamics through crown damage and elevated tree mortality [2], but also because catastrophic branch failure in urban trees severely affects critical human infrastructure. However, despite the potential for more and increasingly severe events with climate warming [3], [4], there have been few attempts at developing an understanding of the ice accretion process in tree crowns.

The often extraordinary effects of ice storms on trees have drawn the attention of researchers for more than a century. Early studies described crown damage from icing qualitatively [5]–[7]. Later studies have concentrated on assessments of tree-level damage to an event using numerous, frequently arbitrarily chosen damage classes [8]–[15]. Only a few papers have quantified the number of fallen branches [16] and the volume of woody litter [13], [17], [18]. Others have related percent canopy damage to spatial variation in radial ice accretion, the latter being the reported maximum radial ice thickness (mm) on a network of horizontal cylinders monitored at neighbouring airports [19]. However, extrapolating this measure to ice accretion on branch elements arranged in a tree crown assumes equivalent radial ice accretion along branches and at all positions within a crown [2], [19]. Similarly, related research on natural snow loading on branches has also subsumed along-branch variation [20]. Surprisingly, despite the long-standing interest in ice storms, the process of ice accretion and thus ice loading on branches remains poorly understood. This is likely because empirical measurements from ice and snow monitoring systems are difficult to extrapolate to branching systems, as well as difficulties hindering *in situ* measurements of ice on branches, including: 1) measuring branches in tall canopies, 2) limitations to travel during and immediately after events 3) the unpredictability of ice storm events, and (4) the fact that the advancing warm front quickly arrives at the local ground level and melts the ice.

In a recent paper, an experimental approach to studying the effects of freezing rain was tested [18]. Trees in two plots in a hardwood forest were treated by simulating freezing rain, resulting in 7–12 mm radial ice thickness [18]. However, efforts were focused on measurements that could be made from the ground: litter fall and changes in canopy openness [18]. Other researchers have also advocated for an experimental approach, in order to determine how ice accretes on branches–i.e. is the radial thickness independent of diameter–so that loading of branches and their biomechanical limits could be estimated [2]. Such an understanding is critical for understanding variation in damage related to species identity and/or position in the canopy.

One expects a bias towards the interception of freezing rain at branch tips, causing them to accumulate ice at a greater rate and leading to a positive feedback; i.e. dependence of the radial ice accumulation rate on branch (plus ice) diameter so that the icing rate decreases from branch tip toward the bole [2]. In addition, variation in position within a canopy on branch ice accretion is often speculated upon in the literature, with lower branches presumably receiving lessened accretion due to the branches above intercepting rain. This speculation has never been quantified to our knowledge. Note how both these issues, the expected decline in icing from branch tip to bole and from the upper to lower crown, cannot be assessed by examination of woody litter or of canopy openness as assessed from the ground.

Given the difficulties associated with accessing the crowns of large mature trees, as well as the challenge of recording data on crown structure in 3D by hand, researchers have sought to utilize methods of remote sensing, such as terrestrial laser scanning (TLS) to measure various aspects of tree crown structure, including branch diameter and geometry [21]. TLS would provide an ideal tool for measuring ice accretion in tall canopies, however, there is little if any research documenting the accretion of ice from freezing rain using measurements from a TLS.

We argue that both an experimental approach rooted in TLS and the development of mechanistic models of accretion and breakage will lead to better prediction of the inter-specific effects of ice storms on eastern deciduous forests. Here we present the first results from an experimental outdoor icing of a mature *Acer platanoides* L. in the city of Montreal. To our knowledge, this is the first study to successfully quantify *in situ* ice accretion on branches, as well as the first to employ TLS to measure ice accretion. Specifically, our goals were to: 1) evaluate the feasibility of experimental icing, 2) test the method of using TLS to capture ice accretion and structural changes in tree crowns, and 3) test the hypothesis that radial ice accretion on branches varies with diameter (distance from branch tip) and with crown position. A subsequent paper will use this data set to test models of accretion and breakage.

## Methods

### Study Site and Species

This study was conducted in the city of Montréal, Québec, Canada. The study species, *A. platanoides*, is the most common urban tree in eastern North America (CA Nock, unpublished data). The individual studied was typical for urban *A. platanoides* street trees, and was situated on a flat site with wide spacing (∼6 m) between neighbouring trees. Permission to utilize the field site and conduct the experiment was granted by The City of Montréal and The Canada Lands Company. Due to the lack of competition the lower crown was much more fully developed than with a forest tree. A one story building was situated ∼5 m to the northeast since the tree was planted, but was demolished a few years prior to the experiment. Tree age was determined from ring counts to be 43 years, diameter at breast height was 35 cm, height was ∼9 m and crown diameter was ∼5 m.

### Simulation of Freezing Rain

Simulation of freezing rain will be influenced by variables such as expected air temperature and its diel variation, water droplet size, falling time, area to cover, flow rate and source water temperature. In this study, our goal was to simulate a “realistic” freezing rain event on branches. This meant that we needed to avoid the formation of icicles (a rare phenomenon in real icing events). We also sought to create a uniform pattern of rain over an area several meters wider than the crown itself.

**Figure 1:**
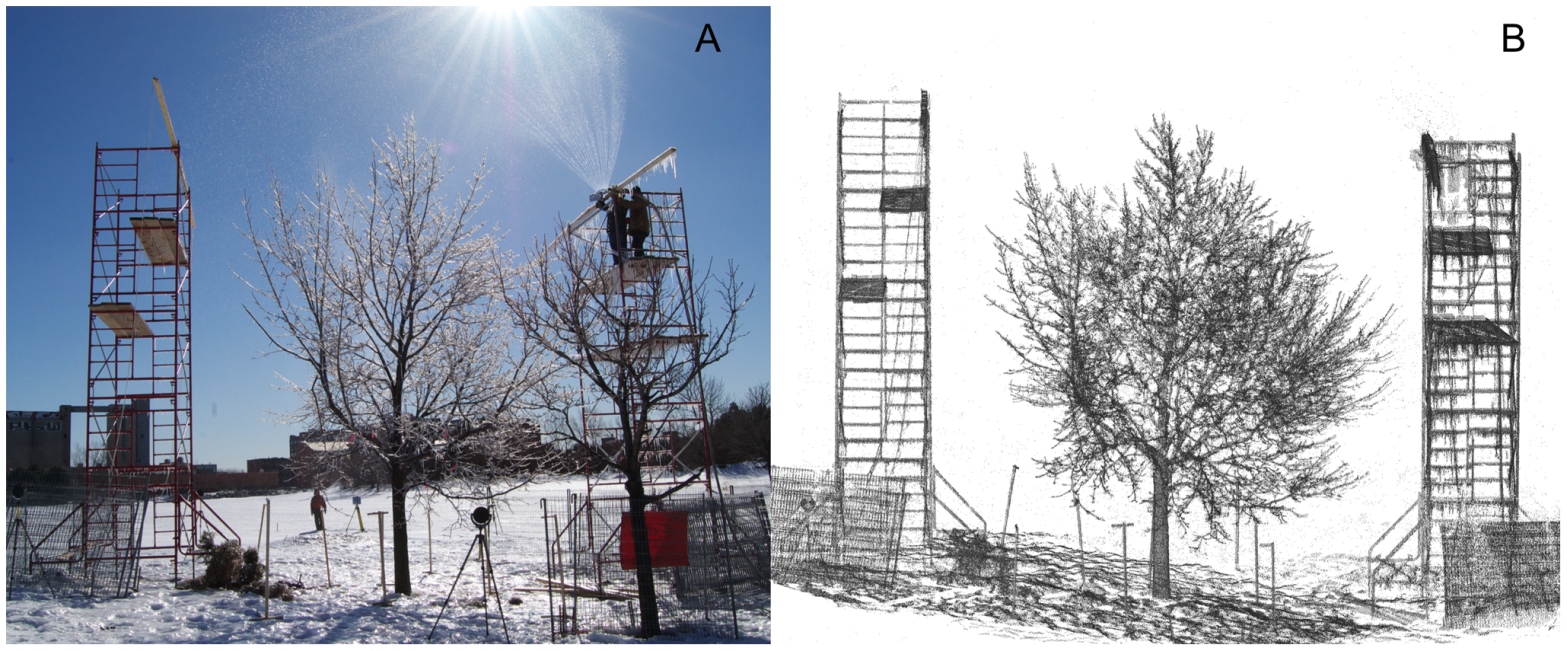
Experimental design with the study tree in the center and scaffolding towers to elevate the sprinkler system (A) and progressive change in tree architecture with increasing ice accretion (B).

Developing an irrigation system required testing methods and observing the shape of ice on branches and the propensity for the water nozzles to freeze at temperatures ranging from −5°C to −20°C. After a series of small-scale outdoor trials in January, we tested two possible irrigation systems on a neighbouring tree ∼15****m away from the experimental tree. We irrigated from a scaffolding tower equal to the height of the tree (∼9 m working height; **Fig. 1**) so that rain fell from a realistic angle for the wind speed i.e. close to perpendicular to the ground. For the first trial we utilized greenhouse irrigation nozzles inserted into flexible plastic poly vinyl piping and strung between the two scaffolds, but the nozzles would ultimately freeze at temperatures below −10°C and water would stream from the frozen nozzles. Further, the ice produced from the fine droplets was white like rime ice (due to trapped air bubbles), which is lower in density than ice formed during freezing rain. In the second trial, we used an oscillating garden sprinkler. We found that it produced larger water droplets more suited to common January-February temperatures and the apertures (1.23 mm in diameter) were mostly resistant to freezing during operation, although one unit did freeze during the experiment and needed to be replaced with a backup (Orbit 3600 oscillating sprinkler, Orbit Irrigation Products, Bountiful, Utah, USA). Water output of the sprinkler was 17 L min^−1^, which given the area covered (∼160 m^2^) converts to 6.15 mm h^−1^ and ∼40 mm of precipitation for the duration of the experiment. The diameter range of water droplets produced was 0.9 mm–2.8 mm and the mean droplet size was 2 mm. This was determined from dusting Whatmans #2 filter paper with methylene blue and exposing the papers to one timed cycle of the oscillating sprinkler. Diameters of the water droplet stains (n = 103) were measured using the program ImageJ [22] and stain diameter converted to rain drop diameter using a published calibration [23].

The experiment was deliberately conducted over two relatively calm days (so that wind-induced drag would not be a contributing cause to breakage, and the irrigation would be confined to a small area) from February 22–23, 2011. For this paper, we only utilize the data for the first day of icing. On that day there was a gentle breeze (3–5 m/s) and temperature ranged from a low of −15°C to a high of −6°C. Fire hoses carried the source water 30 m from a hydrant to the site, where a 1/2” reducer was used to connect to plastic poly vinyl piping and then to the sprinkler. The experimental irrigation began at 9∶30 and ended at 16∶00 (**Table 1**).

**Table 1:** Timing of terrestrial laser scanning scans of the tree during the experiment and average tip ice accretion at a given time.

Scan interval	Hours after irrigation started (actual time)	*Radial ice accretion at branch tips (mm)		Radial ice accretion on dowels	
		*upper*	*lower*	*beyond crown*	*below crown*
0	0/9∶30	–	–	–	–
1	2.5/12∶00	7	7	7	5
2	6.5/16∶00	30	12	15	10

* Note: Radial ice accretion at branch tips taken from the predicted relationship (see Table 3, Figure 5).

### Measuring Tree Canopy Structure and Ice Accretion

We conducted trials to test the abilities of different TLS devices to detect ice on branches using an ILRIS-3D (Optech Inc., Vaughan, Ontario, Canada) and a Z+F laser scanner (Z+F USA Inc., Bridgeville, PA, USA). On a cold day in January a ∼3 m long section of branch was supported at a 45 degree angle by a ladder. An initial ice-free scan of the branch was made. Then a fine mist of water was sprayed on the branch from a garden hose with a misting setting. After an accumulation of 5–10 mm radial ice accretion on branch tips the scene was scanned and the data inspected to determine if the ice coating was detectable. Both, ILRIS and Z+F scans were taken at about 10 m from the branch with medium resolution settings.

Then, during the experiment, we generated two data sets on ice accretion. The first comprised manual measurements that served as validation data, and the second was produced from TLS. Collection of 3D point clouds was acquired using the Z+F device with settings allowing a beam size of 5 mm at 10 m and a distance of 0.34 mm between points at 10 m (for detailed specifications on the Z+F see **Table S1**). Prior to icing and several times during the experiment, the study tree was scanned from three positions separated by ∼120 degrees, each at a distance comprised between 12 and 17 m. We used the high resolution setting and the time to complete a scan was approximately 3 minutes. The multiple scans for the same scanning interval (Table 1) were later registered using the locations of stationary targets and the Z+F software (http://www.zf-laser.com/Home.91.0.html?&L=1; see **Table S2** for registration reports). In some cases targets were obscured due to safety fencing (prohibiting registration in the Z+F software) so fixed points (i.e. intersections of scaffold beams) were used as reference points for scan registration in Pointstream (http://www.arius3d.com/pointstream/). Registration quality was assessed using the alignment of immovable objects in the scans such as the scaffolding, slices of large diameter branches and the main stem.

In parallel, using ladders to reach upper branches, manual measurements were made before icing, at 30 cm intervals along 9 coarse branches (6 upper crown, 3 lower crown) starting at the tips of branches. The locations of these measurements were marked by hanging numbered aluminum tags at the point of measurement, using thin wire, so the same point could be re-measured after icing. Two caliper measurements were made at each point along the branches, perpendicular to each other, with the first being parallel to the ground. This was done at the first scanning of the tree, prior to the onset of irrigation.

In addition to measurements on branches, ice accretion was measured on eight wooden dowels (2.54 cm diameter) positioned parallel to and 1 m above the ground. Four of these wooden dowels were located beneath the crown, halfway between the drip line and the trunk to obtain an estimate of the through-fall intercepted by the canopy. The other four were placed ∼2 m beyond the furthest extant of the canopy. Dowel thickness was measured with calipers after the first and second scanning (**Table 1**). In one case security fencing obscured a dowel during lidar scanning resulting in limited point cloud data.

### Extracting Diameter Measurements from Point Cloud Data

**Figure 2:**
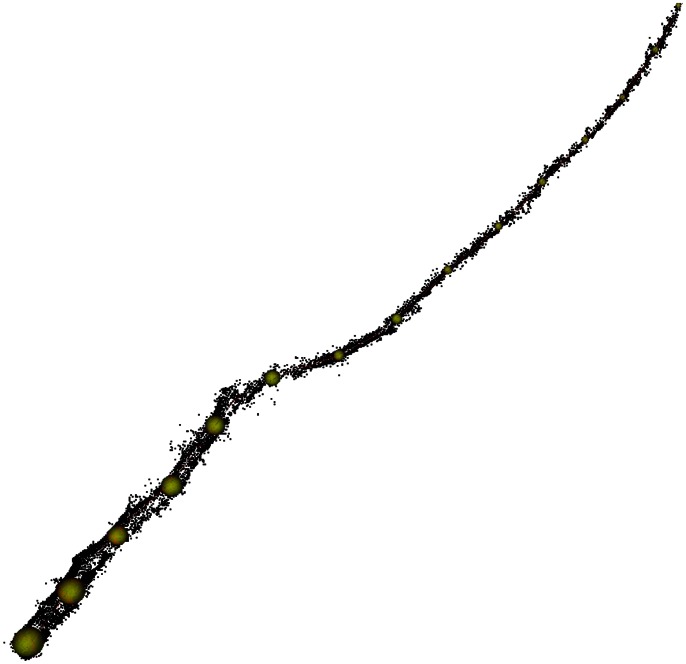
Illustration of the method for measuring diameter of dowels and branches from point cloud data. The diameter of the sphere is determined by fitting cylinders to the point cloud using an algorithm and data is then output for the diameter at each point on the branch marked by a yellow sphere. Sphere diameter is then manually adjusted if visual inspection suggests a better fit is possible. The branch shown has 15 points of measurement at ∼30 cm intervals.

We used PypeTree**,** an open source Python and VTK-based software tool for the reconstruction and modeling of botanical trees from point cloud data (e.g. acquired from TLS devices), to fit a series of cylinders to the 3D point data for each branch (details of the reconstruction process available at http://cjauvin.github.com/pylidar/markers.html). Based on the PypeTree reconstructions, measurement markers (i.e. spheres with a position and radius) are generated at evenly spaced interpolated intervals (30 cm to match validation data). Measurement marker radius is then adjusted if the limits of the markers are smaller or exceed the boundaries of the point cloud at any given location on a branch (**Fig. 2**).

### Radial Equivalent Ice Thickness

Studies of icing on cables and towers use the *equivalent radial ice thickness* (R_eq_) as the standard measure of ice accretion from freezing rain, where R_eq_ is the radius of ice added to a cylinder if the ice was distributed uniformly around the cylinder circumference; i.e. it is the mean added radius. Measurements of R_eq_ were made by randomly collecting samples of ice-covered broken branch elements. For some larger samples branch diameter was determined from the curvature in the ice. Samples were placed in a freezer on-site. Measurements on the geometry were made later along each branch, with R_eq_ calculated as:
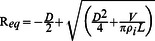
where *D* is the diameter (cm) and *L* is the length of the branch element (cm), *V* is the volume of the melted ice (milliliters) and 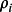 is the density of glaze ice (g cm^−3^) [2], [24].

### Statistical Analyses

To assess the ability of TLS to capture branch architecture and ice accretion, we used linear regression to relate caliper measurements to estimates of branch diameter from measurement markers in PypeTree. Similarly, measurements of equivalent radial ice thickness (R_eq_) were related to caliper measurements of ice radial thickness using linear regression. Finally, multiple linear regression was used to related radial ice accretion to distance from branch tips (and to branch diameter), position in the tree crown and time during the day of data collection. All statistical analyses were conducted in R v.2.15.2 (http://www.r-project.org/).

## Results

**Figure 3:**
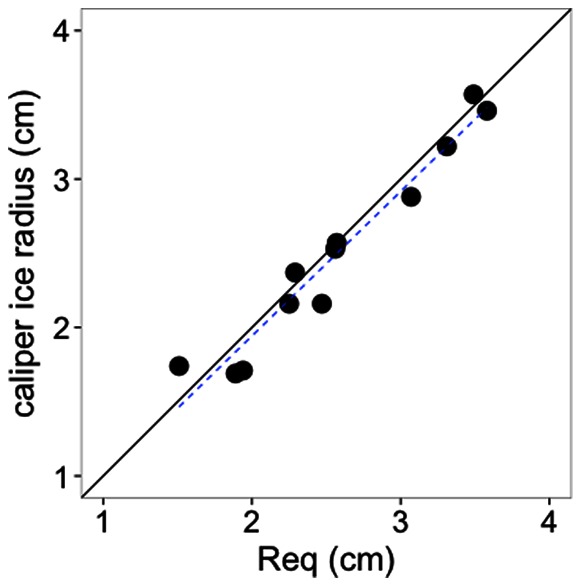
Relationship between equivalent radial ice thickness (R_eq_) and caliper measurements of ice radial thickness. R_eq_ is estimated from the volume of ice (melt water) on branch samples with a given diameter and length. The solid line indicates a 1∶1 relationship and the dotted line the fitted relationship (y = −0.004+0.97x; F = 179.2, DF = 10, p<0.001, R^2^ = 0.95).

Freezing rain produced radial ice accretion averaging 30 mm on branch tips in the upper crown and 7 mm on tips in the lower crown (**Table 1**). The shape of accreted ice on branches was generally cylindrical; incipient icicles occurred but were rare. There was therefore a tight relationship between caliper measurements of ice radius and R_eq_, and the slope and intercept did not differ from 1 and 0 respectively (**Fig. 3**; R^2^ = 0.95, F = 179.2, DF = 10, p<0.001).

### Measurement of Ice Accretion using TLS Point Clouds

**Figure 4:**
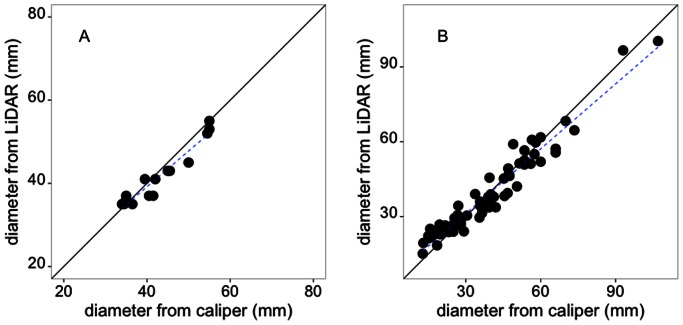
Comparison of the fidelity of terrestrial laser scanning estimates of the diameter of wooden dowels (A) and branches (B) with an ice coating. Branch and dowel diameter was measured using a caliper in the field and diameters for the same locations on branches were also extracted from terrestrial laser scanning point clouds. The solid line indicates a 1∶1 relationship and the dotted line the fitted relationship. Note: Model coefficients and summary statistics presented in Table 2. Timing of terrestrial laser scanning scanning given in Table 1.

The Z+F Imager but not the ILRIS-3D was capable of detecting the ice coating the branches. Estimates of branch and ice diameters produced from measurements of point clouds from TLS closely matched those obtained using a caliper in the field, but with a slight bias toward over-estimation and under-estimation at small and large diameters respectively (**Fig. 4A**). This was evident in the fitted slope for the relationship between caliper and TLS derived diameter, which at a value of 0.87 was slightly less than 1 (**Table 2**; F = 966.4, DF = 64, p<0.001, R^2^ = 0.94). Forcing the x-intercept to zero reduced the bias, leading to a slope of 0.97; for which the 95% confidence interval included 1 (**Table 2**; F = 5159, DF = 65, p<0.001, R^2^ = 0.98).

**Table 2:** Summary of coefficients and 95% confidence intervals for a regression of caliper measurements of diameter (x) versus terrestrial laser scanning measurements of diameter (y) for a subset of measured tree branches and dowels.

Data	Coeff.	Est (SE)		C.I.		t		Pr(>|t|)		F		*RSE		R^2^		DF		p	
*Branches*																			
model 1	Int	5.14 (1.21)	[2.72, 7.56]		4.24		<0.001		966.4		4.26		0.94		64		<0.001		
	Cal	0.87 (0.03)	[0.81, 0.92]		31.09		<0.001												
model 2	Cal	0.97 (0.01)	[0.95, 1.00]		71.82		<0.001		5159.0		4.78		0.98		65		<0.001		
*Dowels*																			
model 1	Int	4.81 (2.99)	[−1.65, 11.27]		−0.52		0.13		156.4		1.97		0.92		13		<0.001		
	Cal	0.86 (0.07)	[0.71, 1.01]		12.51		<0.001												
model 2	Cal	0.97 (0.01)	[0.94, 1.00]		78.43		<0.001		6151		2.08		0.98		14		<0.001		

* Note: RSE is the residual standard error.

For the wooden dowels TLS slightly under-estimated diameter at higher values but the slope and intercept did not differ from 1 or 0 significantly (**Fig. 4B**; **Table 2**; F = 156.4, DF = 13, p<0.001, R^2^ = 0.92). Setting the x-intercept to zero brought the value of the slope to 0.97 and the 95% confidence interval still included 1 (**Table 2**; F = 6151, DF = 14, p<0.001, R^2^ = 0.98).

### Variation in Radial Ice Accretion during the Experiment and with Height in the Crown

For dowels situated below the crown radial ice accretion was similar to lower canopy branches (**Table 1**). For those beyond the limit of the crown, at the second interval radial ice accretion was approximately half of that observed on upper branches (**Table 1**).

**Figure 5:**
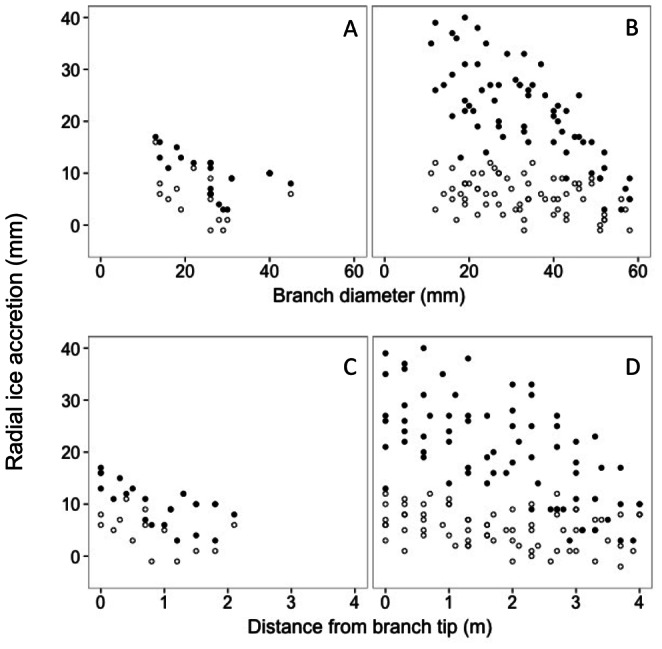
Change in radial ice thickness as measured by increasing distance from branch tips (A, B) or increasing diameter along branches (C, D) for upper (B, D) and lower (A, C) relative positions in the crown. Hollow circles indicate data collected at the first scanning and solid circles the second scanning (details in Table 1). Fitted model coefficients presented in Table 3.

Radial ice accretion was greater on upper branches than on lower branches at the second scanning interval (6.5 hours of icing) but not at the first scanning interval (2.5 hours of icing; **Fig. 5**, **Table 3**; T:P interaction, p<0.001). This was the case for both models using distance from branch tip or branch diameter to describe the position along branches (**Fig. 5**; **Table 3**).

**Table 3:** Summary of coefficients for models describing the variation in radial ice accretion in a tree crown with crown position (P), location on branch (D)* and time during the experimental exposure of the tree to freezing rain (T).

Data	Coeff.	Est (SE)	C.I.	t	Pr(>|t|)	F	RSE	R^2^	DF	p
***Dt***										
	Int	7.18 (1.77)	[3.69, 10.67]	4.06	<0.001	69.3	5.43	0.70	179	<0.001
	T	7.14 (1.86)	[3.48, 10.81]	3.84	<0.001					
	D	−0.79 (1.41)	[−3.57, 1.98]	−0.56	0.575					
	P	0.07 (2.00)	[−3.87, 4.02]	0.04	0.970					
	T:D	−4.30 (0.66)	[−5.61, −2.99]	−6.48	<0.001					
	T:P	15.83 (2.10)	[11.69, 19.98]	7.54	<0.001					
	D:P	−0.19 (1.41)	[−2.97, 2.60]	−0.13	0.894					
***Db***										
	Int	6.47 (2.59)	[1.35, 11.58]	2.49	0.014	94.0	4.86	0.76	179	<0.001
	T	11.19 (1.86)	[7.51, 14.86]	6.01	<0.001					
	D	0.01 (0.09)	[−0.18, 0.18]	0.01	0.998					
	P	2.00 (2.72)	[−3.38, 7.37]	0.73	0.465					
	T:D	−0.31 (0.04)	[−0.38, −0.23]	−7.95	<0.001					
	T:P	15.16 (1.83)	[11.54, 18.78]	8.26	<0.001					
	D:P	−0.08 (0.09)	[−0.26, 0.10]	−0.90	0.371					

* Note: D is described as both increasing distance from the tip (Dt) and with increasing branch diameter (Db). RSE is the residual standard error.

The slope of the change in radial ice accretion along branches–as a function of either branch diameter or distance from branch tips–increased as irrigation proceeded and was significantly greater at the second scanning interval than the first (**Fig. 5**, **Table 3**; T:P interaction, p<0.001). Maximum observed radial ice accretion on upper branches was slightly less than 40 mm, which was close to the 40 mm value for precipitation estimated for the duration of the experiment.

## Discussion

In this study we have demonstrated the feasibility of an experimental approach to understanding ice accretion, employing TLS to overcome the challenges of collecting accurate measurements of radial ice accretion due to the height and 3D complexity of mature tree crowns.

### Methodological Constraints

The design of experimental irrigation systems that are both cost effective and meet design criteria for freezing rain studies must address a number of challenges: a) covering a sufficiently large area of ground with a uniform pattern of drops, b) accessing a source of water during freezing weather, c) producing drops of an appropriate size for the temperature, that freeze soon after contact with trees, and d) producing high enough flow rates so that the equipment does not freeze, or using heating elements or an alternative.

Diel variation in temperature causes significant difficulties for the design of irrigation systems for simulating freezing rain. This is because the risk of equipment freezing increases as water flow decreases, but high rates of water input will lead to excessive flow on the branches at warmer (but still sub-zero) temperatures, creating icicles and other structures that are not commonly observed during natural glaze events and are more difficult to describe with simple geometric shapes (i.e. cylinders). This aspect of irrigation system design may be reflected in the comparison of R_eq_ from this study and from another recent experimental study. For example, in the present study there was a strong relationship between R_eq_ and caliper measurements, whereas in the only previous experimental icing study we are aware of the relationship between caliper measurements and R_eq_ appeared to be weaker–unfortunately, differences in data presentation prevent direct comparison; see Figure 4 in [18]. It is possible that the use of a 1 cm diameter orifice (175 L min^−1^ volume) to spray water up through a gap in the canopy resulted in less regular ice formation [18] and a finer spray, applied over longer time periods would be more realistic [18]. We think that our irrigation design was successful because we could distribute water from above the crown, the sprinkler is designed to distribute the rainfall uniformly and the rate of application of water by lawn sprinklers is moderate. Although it would be more difficult to erect scaffolding in mature hardwood stands, elevation of sprinklers could be achieved using towers or a mobile canopy crane position along an access road. Finally, we emphasize that repeatability is a critical concern when applying any type of treatment in experiments, especially if in future studies the response of forest stands differing in a variable of interest (age, biotic disturbance) to an equivalent freezing rain treatment are to be tested.

### Measurement of Ice Accretion Using Terrrestrial Lidar

TLS provided a novel and accurate method for measuring ice accretion in tree crowns (**Fig. 5**, **Table 3**), permitting us to overcome the challenges of accessing tall canopies and quantifying the multi-dimensional structural complexity of canopy structure. It is important to emphasize that collecting fallen branches and measuring ice accretion on the ground would not have substituted for *in situ* measurements in the *crown–*branches shed ice when they finally snap, again when they may interact with other crown elements as they fall and once again finally when they strike the ground.

In our initial tests we found that not all TLS devices were able to detect ice. For example, ice accumulated on our test branch did not return a signal when we used the ILRIS-3D (no data in scan files). However, as demonstrated here it is certainly not a limitation of TLS in general. Other devices, such as the Z+F employed in the current study, provide a strong signal from ice and snow. This is surely related to the contrasting wavelengths used in both devices as well as to different beam power. Indeed, the ILRIS-3D uses a short wavelength in the infrared range (1535 nm), while the Z+F laser operates in the visible range. Ice and water energy absorption increases significantly in the infrared range while it is very low in the visible [25]. Furthermore, most TLS devices capable of detecting ice utilize class 3 lasers, which are much more powerful than the class 1 laser of the ILRIS-3D. Interestingly, a recent addition to the range of scanners provided by Optech, the ILRIS-LR (http://www.optech.ca/pdf/ILRIS_LR_SpecSheet_110304_Web.pdf, accessed 12/12/2012), utilizes a class 3 laser, has a wavelength closer to visible (1064 nm), and is promoted based on its abilities in detecting snow and ice. We note that operating TLS devices in extreme weather (below −20°C and above 45°C) is not recommended and may represent logistical challenges. One solution is to scan from a vehicle when possible, and we imagine that other methods of protecting the instrument from extreme cold temperatures could be employed.

Irrigation was continued during the scanning process, which required three scan positions separated by ∼120 degrees to prevent ice from forming in water lines. As a result of this limitation we observed some change in the deflection of branches (near tips) in the three different scans, undoubtedly caused by ice loading. This primarily affected branch tips because of their greater flexibility and the apical concentration of stress. However, in most cases, the point cloud density for the side profile of the branch was very similar in at least two scans, and ultimately provided accurate measurements of the diameters of branches with ice compared to measurements made with a caliper in the field (assuming cylindrical branches; **Fig. 4**, **Table 2**). In addition, the relatively quick scanning time of the Z+F (∼10 minutes) minimized the issue. The slight tendency toward under-prediction may have been due to the use of the side profile of branches and deflection of the branch between scans because irrigation was continuous. In future experiments design changes could be made to allow for a cessation of irrigation between scans without water freezing in the irrigation system, for example, rainfall could simply be directed away from the study area between scans or heating elements could be used on the lines.

### Variation in Ice Accretion: Throughfall and within-crown Patterns

In over a century of research on the effects of freezing rain on trees and forests, the most important response variable–the quantity of ice on branches and its variation in the crown–has rarely been quantified due to a number of challenges. To develop an understanding of the processes involved in determining ice loads and ultimately crown damage it is critical to develop an understanding of the variation in ice accretion within tree crowns. While we are not aware of any studies quantifying variation in ice accretion in tree crowns or measuring tree icing with TLS, a few studies have examined loading with icing experimentally or quantified snow and ice loading in the field. For example, in a study of the biomechanics of ice loading and species crotch properties, individual branches attached to a vertical support beam were experimentally iced to examine the branch strength of a number of species [26]. Naturally occurring snow and ice loads (rime) on 10-year-old *Picea sitchensis* have been quantified, but to simplify field data collection snow and ice loads were assumed to be constant per unit length of shoot [20].

We found that ice accretion was very similar for dowels placed beneath the crown and lower canopy branches, with branch tips having slightly higher ice accretion. This reflects the interception of rain drops by branch elements superior to lower branches, with dowels and lower branches exhibiting similar accretion, likely because of similar coverage by superior branches. Based on the same principles and given a uniform pattern of rainfall over the tree and the area beyond it where wooden dowels were placed, we expected ice accretion on upper branches and dowels beyond the crown to be very similar, and while they matched well at the first scanning time, ice accretion on the dowels was lower by ∼15 mm at the second scanning time point (**Table 1**). This could have been an effect of a bias toward the center of the watering area of the sprinkler, and/or the beyond crown dowels may have still been influenced by the crown despite the 2 m distance. Consistent with this idea is that ice accreted on the crown would positively reinforce any initial difference through time.

While in previous studies loading has been considered to be independent of branch diameter and/or position along a branch or within a crown [2], [20], it is evident from the present study that ice accretion (radial ice thickness) is highly variable within tree crowns along branches and among branches at different positions within a crown (**Fig. 5**, **Table 3**). While not quantified here, we expect this to depend on factors such as crown depth and crown radius, as well as descriptors of branching intensity such as branch area density.

### Future Research

Vertical variation in ice accretion in tree crowns likely arises because raindrops are intercepted by branch elements in a superior position, so that the probability of a raindrop being intercepted by a canopy element increases with the cumulation of branch area index (m^2^/m^2^, analogous to leaf area index) along a vertical path through the canopy. To develop this conceptual model of ice accretion for tree crowns, one could adapt ray-tracing light models from existing functional-structural models, but simply fix the angle of approach (i.e. raindrops as photons that stick to branches instead of scattering). Alternatively, one could also employ voxel-based analysis and attempt to relate variation in ice accretion on branches with counts of voxels along transects through the canopy. Finally, the data generated on ice accretion and branch allometry in the present study will permit detailed modeling of the stresses on branches with additional information on material properties (e.g. [20]).

We echo the call for new experimental approaches to understanding the effects of disturbances such as ice storms [18]. Our approach could be applied at the stand level and in a more natural setting (e.g. [18]). Experimenting on still larger trees in a forest setting would likely not be possible using scaffolding, but could be possible using alternative methods to access canopies at even greater heights.

## Supporting Information

Table S1 **A table showing the technical specifications for Z+F imager 5006i.**
(DOCX)

Table S2 **Registration reports for the alignment of the TLS scans from the Z+F laser scanner.**
(DOCX)
