# Supplementary material for: In Situ Quantification of Experimental Ice Accretion on Tree Crowns Using Terrestrial Laser Scanning
Source: PLoS One. 2013 May 31;8(5):e64865. doi: 10.1371/journal.pone.0064865 (PMC3669131; doi:10.1371/journal.pone.0064865)
Supplement: Table S1 — A table showing the technical specifications for Z+F imager 5006i. (DOCX) [file pone.0064865.s001.docx]

**Table S1.** Technical specifications for Z+F imager 5006i

| **Item** | **Value** | |
| --- | --- | --- |
| ***Laser measurement system*** |  |  |
| Ambiguity interval | 79 m  0.4 m  0.1 mm  < 508 000 pxl/sec.  < 1 mm | |
| Min. range |  |  |
| Resolution range |  |  |
| Data acquisition rate |  |  |
| Linearity error up to 50 m^1^ |  |  |
| Range noise at 10 m^1,2^ |  |  |
| Reflectivity 10% (black) | 1.2 mm rms  0.7 mm rms  0.4 mm rms | |
| Reflectivity 20% (dark grey) |  |  |
| Reflectivity 100% (white) |  |  |
| Range noise at 25 m^1,2^ |  |  |
| Reflectivity 10% (black) | 2.6 mm rms  1.5 mm rms  0.7 mm rms | |
| Reflectivity 20% (dark grey) |  |  |
| Reflectivity 100% (white) |  |  |
| Range noise at 50 m^1,2,3^ |  |  |
| Reflectivity 10% (black) | 6.8 mm rms  3.5 mm rms  1.8 mm rms | |
| Reflectivity 20% (dark grey) |  |  |
| Reflectivity 100% (white) |  |  |
| Range drift over temp. (-10°C to 45°C) | Negligible due to internal reference | |
| ***Optical transceiver*** |  |  |
| Laser | visible  0.22 mrad  3 mm circular  3R (ISO EN 60825-1) | |
| Beam divergence |  |  |
| Beam diameter at 1 m distance |  |  |
| Laser safety class |  |  |
| ***Deflection unit*** |  |  |
| System vertical | Rotating mirror  Rotating device  310°  360°  0.0018°  0.0018°  0.007° rms  0.007° rms  < 50 rps  25 rps | |
| System horizontal |  |  |
| Field of view vertical |  |  |
| Field of view horizontal |  |  |
| Resolution vertical |  |  |
| Resolution horizontal |  |  |
| Accuracy vertical^1^ |  |  |
| Accuracy horizontal^1^ |  |  |
| Max. scanning speed vertical |  |  |
| Typ. Scanning speed vertical |  |  |
| ***Resolution*** |  |  |
| *Resolutions* | *Pixel / 360°* | *Scanning time / 360°* |
| “preview” | 1250 | 25 sec |
| “middle” | 5000 | 1 min 40 sec |
| “high” | 10000 | 3 min 22 sec |
| “super high” | 20000 | 6 min 44 sec |
| “ultra high” | 40000 | 26 min 40 sec |
| Max. resolution for selections | 100000 | - |

^1^Contact [imager5006@zf-laser.com](mailto:imager5006@zf-laser.com) for more information. ^2^Data acquisition rate: 127000 pxl/sec., raw data, in high power mode. ^3^Values extrapolated.
