# Supplementary material for: In Situ Quantification of Experimental Ice Accretion on Tree Crowns Using Terrestrial Laser Scanning
Source: PLoS One. 2013 May 31;8(5):e64865. doi: 10.1371/journal.pone.0064865 (PMC3669131; doi:10.1371/journal.pone.0064865)
Supplement: Table S2 — Registration reports for the alignment of the TLS scans from the Z+F laser scanner. (DOCX) [file pone.0064865.s002.docx]

**Table S2.** Registration reports for the alignment of scans from a Z+F laser scanner.

*Ice free*

| Enabled targets whose deviations are greater than the defined threshold 7.0 mm | 14 of 17 |
| --- | --- |
| Total number of targets | 18 |
| Number of disabled targets | 1 |
| Average Deviation | 10.1 mm |
| Standard Deviation | 4.6 mm |
| Maximal Deviation | 17.5 mm |

*Scanning interval 1*

| Enabled targets whose deviations are greater than the defined threshold 7.0 mm | 6 of 13 |
| --- | --- |
| Total number of targets | 15 |
| Number of disabled targets | 2 |
| Average Deviation | 15.3 mm |
| Standard Deviation | 10.0 mm |
| Maximal Deviation | 32.5 mm |

*Scanning interval 2*

| Enabled targets whose deviations are greater than the defined threshold 7.0 mm | 4 of 15 |
| --- | --- |
| Total number of targets | 16 |
| Number of disabled targets | 1 |
| Average Deviation | 8.1 mm |
| Standard Deviation | 5.7 mm |
| Maximal Deviation | 19.9 mm |
